# Contribution of researchers in Arab countries to scientific publications on neglected tropical diseases (1971 – 2020)

**DOI:** 10.1186/s40794-022-00173-7

**Published:** 2022-06-01

**Authors:** Waleed M. Sweileh

**Affiliations:** Department of Physiology and Pharmacology/Toxicology, College of Medicine and Health Sciences, An-Najah National University, Nablus, Palestine

**Keywords:** Neglected tropical diseases, Bibliometric analysis, Arab countries

## Abstract

**Background:**

The neglected tropical diseases (NTDs) are endemic in several Arab countries. The purpose of the current study was to assess the contribution of researchers in Arab countries to the knowledge base on NTDs using bibliometric indicators.

**Methods:**

Keywords related to all 20 NTDs were obtained from previously published bibliometric studies and were combined with the names of Arab countries listed as country affiliation. the search strategy was implemented in the Scopus database and bibliometric indicators were generated for the study period from 1971 to 2020

**Results:**

The search strategy generated 6542 documents; representing less than 4% of the global research in the field. Scientific research on NTDs from researchers in Arab countries (a) has experienced slow growth; (b) generated a relatively inadequate number of publications over the study period; (c) was disseminated mainly through journals in the field of parasitology or tropical medicine; (d) was contributed by researchers from the 22 Arab countries, but mainly by researchers from Egypt, Saudi Arabia, and Sudan; (e) has fragmented author networks with weak collaboration between active authors in the field; (f) was characterized by strong cross-country research collaboration with researchers in the US and the UK; (g) has focused on three main diseases, specifically, schistosomiasis, leishmaniasis, and onchocerciasis, and (h) showed less emphasis on soil-transmitted helminthiasis infections despite high prevalence.

**Conclusions:**

Arab countries cannot achieve the 2030 global agenda without control and elimination of prevalent NTDs. Researchers in Arab countries need to establish strong research networks to exchange expertise on all NTDs.

**Supplementary Information:**

The online version contains supplementary material available at 10.1186/s40794-022-00173-7.

## Background

Neglected tropical diseases (NTDs) are a diverse group of diseases that affect more than one billion people globally, especially in poor regions with tropical and subtropical climates and suffering from poor sanitation and inadequate health services [1, 2]. In contrast to HIV/AIDS, tuberculosis, and malaria, the NTDs receive relatively lesser research funding for the prevention and control [3]. Currently, the World Health Organization (WHO) recognizes 20 conditions, diseases, and disease groups as NTDs [4]. The list includes infectious and non-infectious diseases such as ectoparasites, foodborne trematodiasis, leishmaniasis, soil-transmitted helminthiasis (STH,) and snakebite envenoming (Supplementary Material 1) [5]. The main vector for the transmission of most NTDs is mosquitoes and flies carrying a parasite, bacterium, or virus [6].

The Millennium Development Goals (MDGs), adopted in 2000, focused on fighting major global health problems, the three big killers, including tuberculosis, HIV/AIDS, and Malaria. The MDGs ended in 2015 and were followed by Sustainable Development Goals (SDGs) in which the NTDs received more focus and were mentioned alongside the major health killers [7]. The SDG-03 goal (good health and well-being) states in target 3.3 that “*by 2030 end the epidemics of AIDS, tuberculosis, malaria, and neglected tropical diseases, and combat hepatitis, water-borne diseases and other communicable diseases*”. The NTDs roadmap published by WHO as well as the SDGs played a positive role in increasing attention and research on NTDs [4, 8].

The Arab region includes the 22 countries that form the Arab League, which was created to unify the Arab countries politically [9]. The Arab countries are located in Asia and Africa, with a total area of five million square miles and more than 420 million inhabitants, 6% of the estimated seven billion-world population (Supplementary Material 1). Egypt is the most populous with more than 100 million inhabitants while Comoros is the least populous with less than one million inhabitants. The Arab region has witnessed several conflicts and violent events that generated millions of internally displaced people and refugees living in poverty and limited health services. Yemen, Syria, Libya, and Gaza (Palestinian territories) still suffer from ongoing political instability and internal conflicts. Except for a few Arab Gulf countries, most countries in the Arab region are classified in the low-income category [10]. Poverty, geographical location, political instability, and fragile health system [11, 12] are major risk factors for NTDs in the Arab region [13–16]. In addition, annual religious mass gatherings in Saudi Arabia and Iraq facilitate the spread of NTDs since millions of people from different world regions attend these annual gatherings annually [14].

The Arab region as well as other parts of the world was highly affected by the COVID-19 pandemic leading to disruption of NTD health services [17–22]. Delays in mass drug administration and screening activities of NTDs due to COVID-19 lockdown might lead to a resurgence in certain NTDs [23]. The COVID-19 pandemic has shifted research agendas globally toward virology and development of safe and effective vaccines for COVID-19. The Arab world is no exception and many researchers and research institutions in the Arab countries were affected by COVID-19 due to lockdown, shifting of financial resources, slow supply chain of research materials, and fear of the infection [24]. The disruption of NTD health services and research activity at national and global levels will delay the achievement of the public-health goals set for NTDs [25].

Quantitative analysis of research activity is a powerful approach for revealing global, regional, and country-level efforts to fight NTDs and achieve SDGs. Furthermore, bibliometric analysis help identifies hot research topics and research gaps in the field. Global, regional, and country-level research on NTDs as a group or as a specific NTD showed an increasing trend [26–28]. In this context, the author did a bibliometric analysis of research publications on NTDs in which at least one author is based in the Arab region to assess the extent of contribution of researchers in the Arab region to the field which can be used for future health planning, research funding, and establishing new research collaborations.

## Methods

### Source of information

Several academic databases can be used to obtain bibliometric information. In the current study, SciVerse Scopus was used to retrieve the relevant documents and achieve the objectives. Scopus is a bibliographic database owned by Elsevier (www.elsevier.com) and started in 2004 [29]. Scopus is considered the largest collection of citations and abstracts from peer-reviewed literature and covers multiple fields including arts and humanities, social science, technology, health, and life sciences. Scopus covers approximately 22,800 active titles from more than 5000 international publishers. Scopus is 100% inclusive of MEDLINE journals and almost double the number of journals indexed in Web of Science [30, 31]. Scopus has effective search engine and operating functions that facilitate analysis such as journal name, year of publication, type of the document, open access, authors and country affiliation, Scopus has a document search service that allows for building complex search strings using different Boolean operators such as ‘AND’, ‘OR’, and ‘NOT’ with specific keywords to generate a comprehensive query for obtaining relevant data. That is why Scopus was used in many previously published health-related bibliometric studies [32–34].

### Ethics approval

The current study did not involve any human subject participation. Method and results were entirely performed using data retrieved from Scopus. Therefore, the Institutional Review Board considered the current study to be exempted from ethics approval procedure.

### Study design

The current study was a cross-sectional descriptive bibliometric review analysis that used bibliometric analysis to monitor research performance on NTDs to support future policy actions.

### Search string

The search string and strategy was developed and validated by the author. The search string was developed based on keywords used in previously published bibliometric studies on NTDs [27, 35, 36]. The current study used the title search strategy rather than the title-abstract search strategy to maximize accuracy and avoid false-positive results. To limit the search strategy to the Arab region, the names of the 22 Arab countries were listed in the strategy as country affiliation. The keywords used for the 20 NTDs and the full search strategy are shown in Supplementary Material 2.

The search strategy was limited to the study period from 1971 to December 31, 2020, representing half a century of research activity. No language restriction was used in the search strategy. However, only documents published in scientific peer-reviewed journals were included. Furthermore, only documents that are published in the final stage were included.

### Data export and analysis

The retrieved data were exported into Microsoft Excel for analysis and mapping. The analysis focused on descriptive analyses, such as the frequencies of the types of documents, the language, open access, annual number of scientific publications, subject areas, names of leading journals, countries, and institutions. The second part of the analysis was the bibliometric mapping using the VOSviewer software [37] to generate co-occurrences of author keywords, overlay visualization, co-authorship network, and cross-country collaboration network. In author keyword co-occurrences, the units of analysis are author keywords. The units of analysis are represented in the maps as circular nodes. The size of the node accounts for the frequency of occurrences and the position represents the similarity with other nodes in the map. Closer nodes are more alike than nodes far apart from each other. The lines connecting nodes represent the relationship between nodes and their thickness indicates the strength of that relationship. Finally, the color of the node denotes the cluster to which each node has been allocated. Nodes are clustered together based on relatedness. In the overlay visualization map, the map of author keywords co-occurrences is presented based on the time (year of publication) of appearance the keyword in the literature. Co-authorship mapping was performed to examine the social structure of research. In co-authorship mapping, the units of analysis were authors. Each node in the map represents an author and the lines connecting them reflect the relationship between nodes. Clusters represent networks of scientific collaboration, which might be interpreted as groups of authors frequently publishing together (e.g., research groups of authors). Cross-country collaboration mapping was performed to examine the international research collaboration. The units of analysis were countries. The size of the node represents the number documents with international authors. Countries with large node size have large number of documents with international authors. The VOSviewer uses the term “total link strength = TLS” to represent the extent of international research collaboration with higher TLS values associated with large node size. The TLS is proportional to the number of links coming out of the node representing the country. The thickness of a connecting line between two countries represents the strength of research collaboration between the specified countries (number of documented with authors from the two specified countries). Similar colors of the nodes represent countries with related research interests.

## Results

### General characteristics of the retrieved documents

The search strategy found 6542 documents on NTDs published from 1971 to 2020 with at least one author affiliated with one Arab country. There were several types of retrieved documents, mainly research articles (*n* = 5,978; 91.4%). Table 1 shows all types of retrieved documents. There were 548 (8.4%) documents with bilingual abstracts (English and non-English abstracts). The remaining documents (*n* = 5994; 91.6%) were unilingual using English language only. The retrieved documents belonged to different subject areas mainly medicine (*n* = 5144, 78.6%), immunology/microbiology (*n* = 2083, 31.8%), agricultural and biological sciences (*n* = 746, 11.4%), biochemistry/molecular biology (665, 10.2%), and veterinary (*n* = 514, 7.9%).

**Table 1:** Types of the retrieved documents

Type	Number of documents	% (*N* = 6542)
Article	5978	91.4
Review	261	4.0
Letter	183	2.8
Note	43	0.7
Conference Paper	42	0.6
Short Survey	19	0.3
Editorial	16	0.2

### Annual growth of publications

**Fig. 1:**
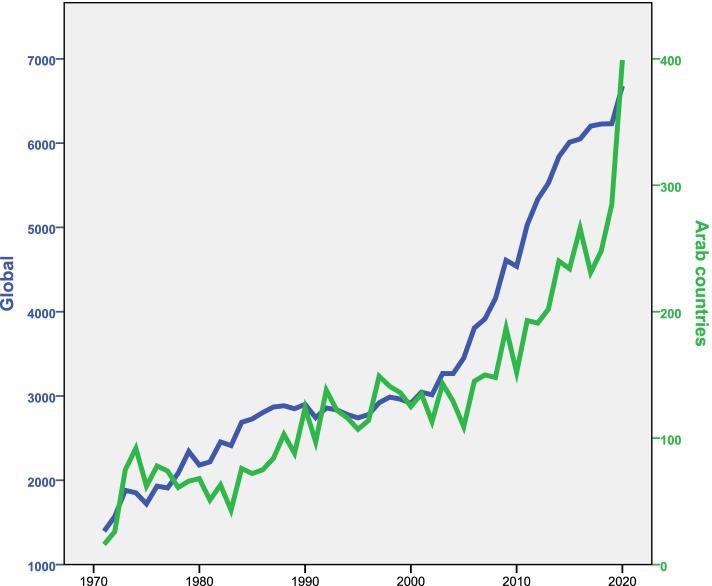
Annual growth of publications on NTDs by researchers in Arab countries and worldwide (1971 – 2020)

Analysis of the retrieved data showed slow and gradual growth in the number of publications over time (Fig. 1). The number of publications from 2016 to 2020 represented more than one-fifth (*n* = 1429; 21.8%) of the total number of publications. The number of publications on NTDs produced globally during the same study period was 170,398. Therefore, researchers in the Arab region contributed to 3.8% of the total research publications on NTDs produced globally.

### Geographical distribution of the retrieved documents

**Fig. 2:**
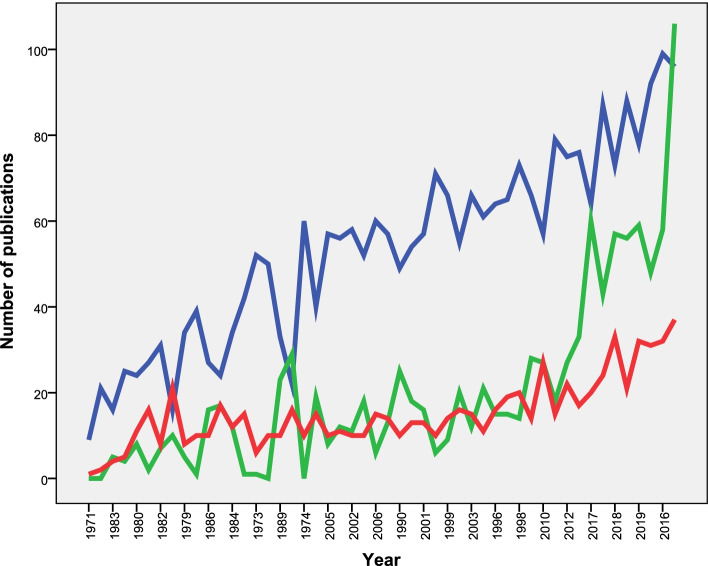
Annual growth of publications on NTDs by researchers in Egypt (Blue), Saudi Arabia (Green), and Sudan (Red) (1971 – 2020)

Researchers from the 22 Arab countries contributed to the retrieved documents (Table 2). However, researchers from Egypt (*n* = 2677; 40.9%) made the most contribution followed by Saudi Arabia (*n* = 1019; 15.6%), Sudan (*n* = 758; 11.6%), and Tunisia (*n* = 597; 9.1%). Figure 2 shows the annual growth of publications from the top three Arab countries. The annual growth of publications from Saudi Arabia and Sudan was close until 2010 followed by a steep growth of publications from Saudi Arabia that exceeded that from Sudan and Egypt. The analysis also showed that academic and research institutions in Egypt and Saudi Arabia dominated the list of top 10 active institutions (Table 3). The United States (US) (*n* = 608; 9.3%) was the most collaborative country with researchers in the Arab countries followed by the United Kingdom (UK) (*n* = 469; 7.2%).

**Table 2:** Contribution of each Arab country and the top 10 collaborating non-Arab countries

**Arab country**	**Number of documents**	**% (*N*** **= 6542)**
Egypt	2677	40.9
Saudi Arabia	1019	15.6
Sudan	758	11.6
Tunisia	597	9.1
Morocco	439	6.7
Iraq	316	4.8
Algeria	172	2.6
Kuwait	150	2.3
Jordan	126	1.9
Yemen	126	1.9
Lebanon	111	1.7
Oman	96	1.5
Libyan Arab Jamahiriya	89	1.4
United Arab Emirates	86	1.3
Qatar	59	0.9
The Syrian Arab Republic	54	0.8
Palestine	50	0.8
Somalia	31	0.5
Mauritania	27	0.4
Bahrain	22	0.3
Comoros	8	0.1
Djibouti	4	0.1
**Non-Arab country**	**Number of documents**	**% (*N* = 6542)**
United States	608	9.3
United Kingdom	469	7.2
France	306	4.7
Germany	183	2.8
Switzerland	177	2.7
India	157	2.4
Netherlands	152	2.3
Italy	124	1.9
Belgium	98	1.5
China	83	1.3

**Table 3:** Top 10 active institutions

Institution	Number of documents	% *N* = 5642	Country Affiliation
*Cairo University*	509	7.8	Egypt
*Khartoum University*	449	6.9	Sudan
*Alexandria University*	315	4.8	Egypt
*Theodor Bilharz Research Institute*	296	4.5	Egypt
*Ain Shams University*	443	6.8	Egypt
*King Saud University*	259	4.0	Saudi Arabia
*National Research Centre*	217	3.3	Egypt
*Mansoura University*	189	2.9	Egypt
*Université de Tunis El Manar, Institut Pasteur de Tunis*	212	3.2	Tunisia
*King Abdulaziz University*	138	2.1	Saudi Arabia

### Prolific journals

**Fig. 3:**
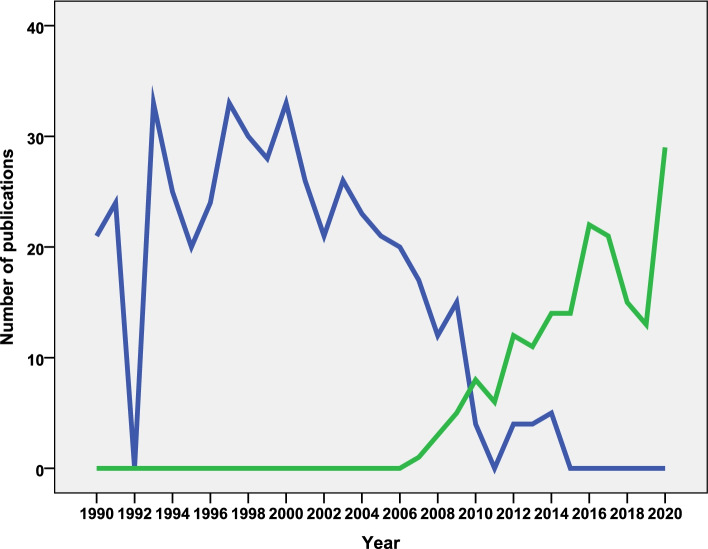
Annual growth publications on NTDs by researchers in Arab countries in the *Egyptian Society of Parasitology* journal (Blue) the *Plos Neglected Tropical Diseases* journal (Green) (1971 – 2020)

The retrieved documents were disseminated through 1486 scientific journals. The *Journal of the Egyptian Society of Parasitology* was the most prolific (*n* = 497; 7.6%) followed by *Transactions of the Royal Society of Tropical Medicine and Hygiene* journal (*n* = 241; 3.7%). Table 4 shows the names of scientific journals, which published 50 or more documents on NTDs with at least one author from Arab countries. The list included 14 journals that published 1919 (29.3%) documents. The *Journal of the Egyptian Society of Parasitology* was the favorite journal for researchers in the Arab region up to 2010 while the *Plos Neglected Tropical Diseases* was the favorite for Arab researchers from 2010 to 2020 (Fig. 3).

**Table 4:** List of journals with at least 50 publications on NTDs with authors from Arab countries (1971 – 2020)

*Journal*	Number of documents	% (*N* = 5642)
*Journal Of the Egyptian Society of Parasitology*	497	7.6
*Transactions Of the Royal Society of Tropical Medicine and Hygiene*	241	3.7
*American Journal of Tropical Medicine and Hygiene*	178	2.7
*Plos Neglected Tropical Diseases*	174	2.7
*Annals Of Tropical Medicine and Parasitology*	136	2.1
*Parasitology Research*	133	2.0
*Acta Tropica*	127	1.9
*Journal Of Helminthology*	77	1.2
*International Journal of Dermatology*	71	1.1
*Parasites And Vectors*	65	1.0
*Journal Of Tropical Medicine and Hygiene*	62	0.9
*Saudi Medical Journal*	56	0.9
*Experimental Parasitology*	52	0.8
*Eastern Mediterranean Health Journal*	50	0.8

### Bibliometric mapping (author keyword co-occurrence)

**Fig. 4:**
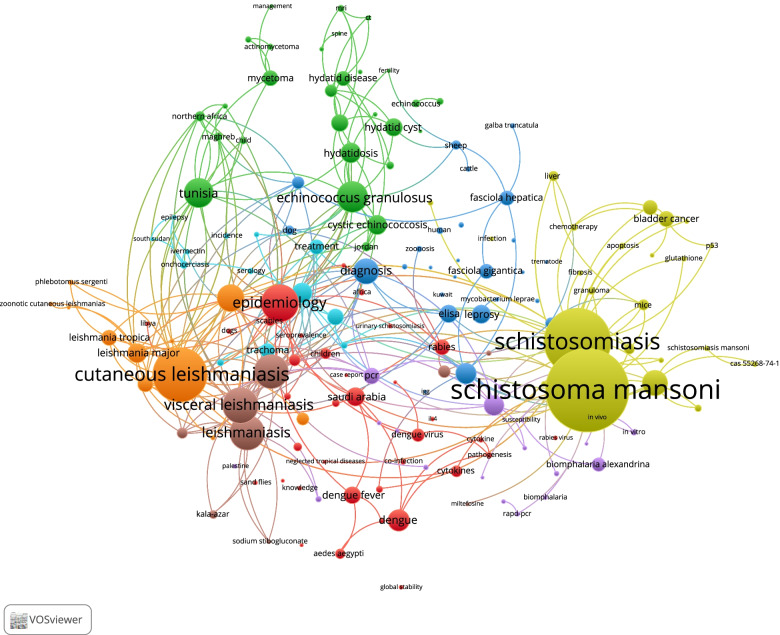
VOSviewer mapping of author keywords with minimum occurrences of 10 in the retrieved documents. Node size is proportional to the frequency of occurrence

**Fig. 5:**
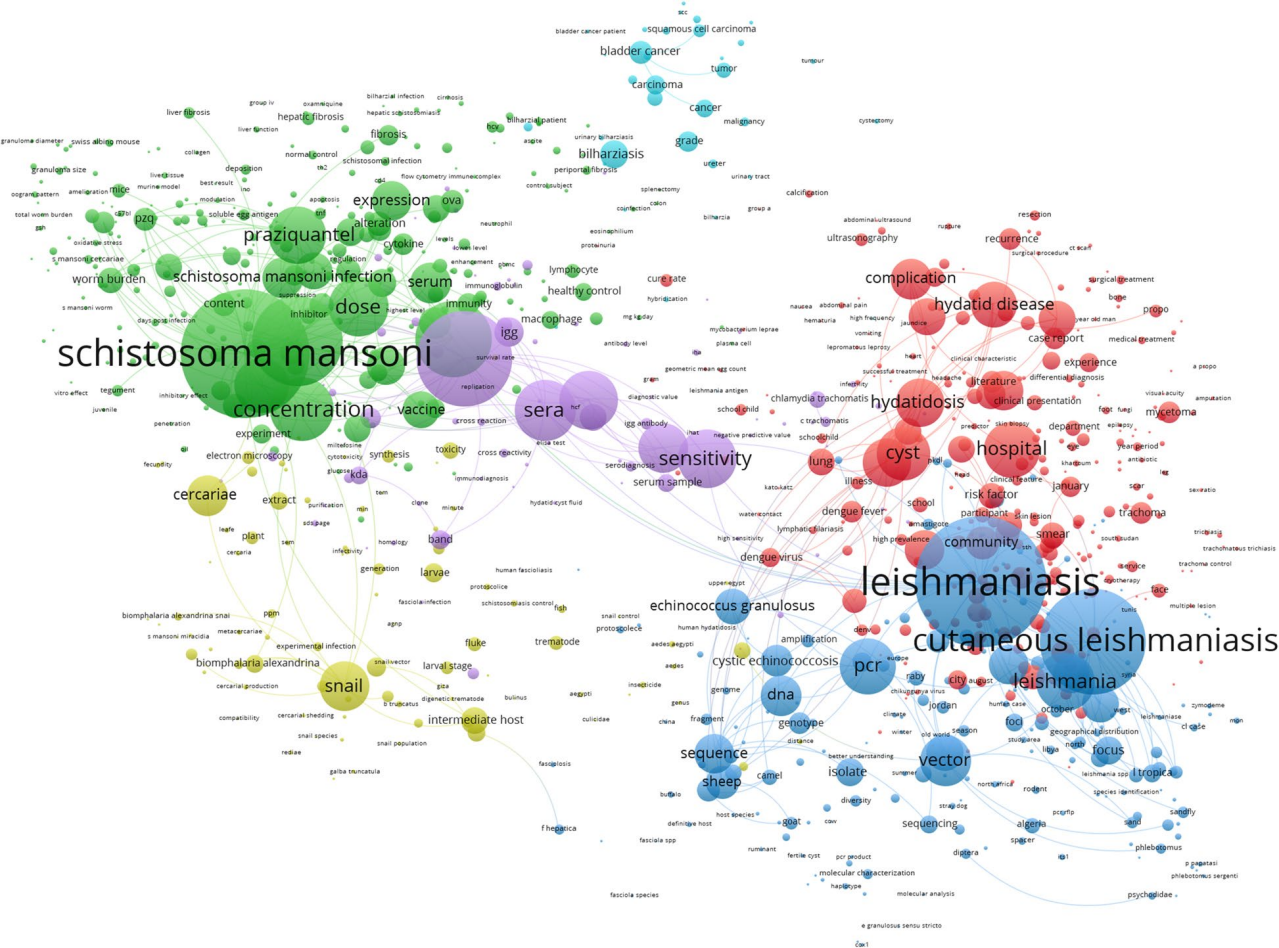
VOSviewer mapping of terms in the titles/abstracts with minimum occurrences of 10 in the retrieved documents. Nodes with similar colors represented related research theme

The VOSviewer mapping of author keywords with minimum occurrences of 10 (Fig. 4) shows that research on schistosomiasis, leishmaniasis, and echinococcosis was the most frequent type of NTDs researched by authors in Arab countries based on the node size. The VOSviewer mapping of terms in the titles/abstracts with a minimum occurrence of 10 (Fig. 5) shows six clusters, each with a different color. The largest cluster represented research on schistosomiasis and was closely adjacent to clusters related to bladder cancer and detection/diagnostic methods of schistosomiasis. The map also shows two large clusters, one on hydatidosis and one on leishmaniasis.

Analysis of the retrieved documents based on the type of NTDs showed that more than one-third of the retrieved documents were about schistosomiasis while approximately one-fifth were about leishmaniasis. Documents on schistosomiasis and leishmaniasis constituted more than 50% of the retrieved documents (Table 5). Buruli ulcer, Chagas disease, and yaws were the least researched by Arab researchers.

**Table 5:** list of NTDs and the volume of research on each disease

Type	Number of documents	% (*N* = 6542)
Buruli ulcer	6	0.1
Chagas disease	24	0.4
dengue and chikungunya	331	5.1
dracunculiasis	26	0.4
echinococcosis	808	12.4
foodborne trematodiases	345	5.3
human African trypanosomiasis	88	1.3
leishmaniasis	1306	20.0
leprosy	212	3.2
lymphatic filariasis	86	1.3
mycetoma and chromoblastomycosis and other deep mycoses	154	2.4
onchocerciasis	70	1.1
rabies	143	2.2
scabies and other ectoparasites	96	1.5
schistosomiasis	2387	36.5
snakebite envenoming	30	0.5
soil-transmitted helminthiases	94	1.4
taeniasis/cysticercosis	43	0.7
trachoma	268	4.1
yaws	25	0.4
NTDs in general	41	0.6
**Total**	**6583**	**100.6**

### Bibliometric mapping (international research collaboration)

**Fig. 6:**
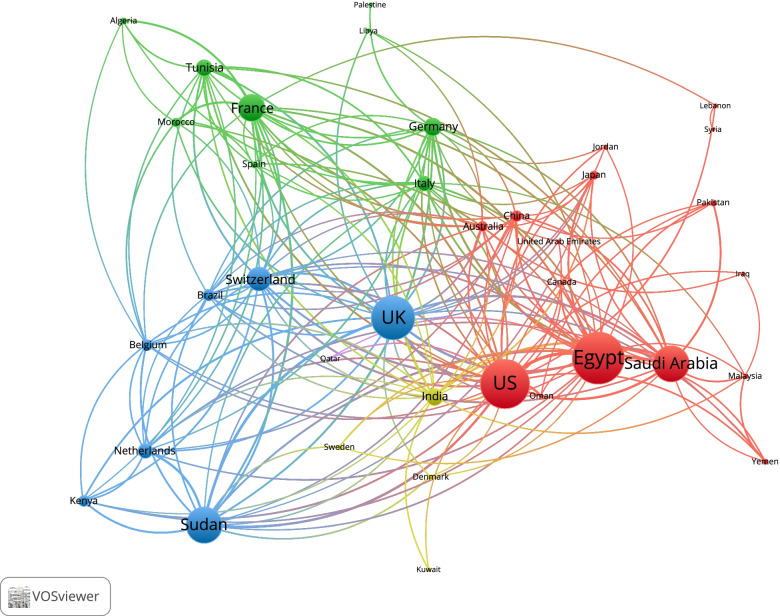
VOSviewer mapping of cross-country (international) research collaboration among countries with a minimum contribution of 50 documents. Node size is proportional to the number of documents from the specified country with international authors. Thickness of the connecting line denotes strength of international research collaboration between the connected countries

The VOSviewer mapping of cross-country (international) research collaboration among countries with a minimum contribution of 50 documents shows 37 countries (17 Arab and 20 non-Arab countries) (Fig. 6). The strongest research collaboration was between the US and Egypt (TLS = 286), followed by that between Egypt and Saudi Arabia (TLS = 186), and between Sudan and the UK (TLS = 166). The map included three large clusters. The largest cluster (red cluster) included eight Arab countries and the US. The blue cluster included five countries and several European countries, mainly France, Germany, and Italy. The blue cluster included one Arab country and the UK. The remaining Arab countries such as Qatar and Kuwait existed in small clusters with weak international research collaboration.

### Prolific authors

**Fig. 7:**
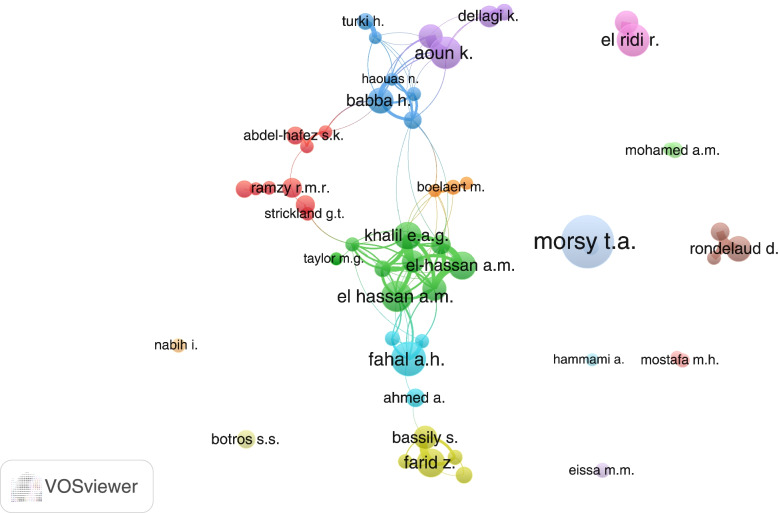
VOSviewer mapping of research collaborations among researchers with a minimum of 20 publications. Authors with similar colors have close research collaboration and represent a cluster

Table 6 shows authors with the highest number of publications in the field. El-Hassan, A.M. appeared to be the most active in this field (*n* = 86; 1.3%), followed by Morsy, T.A. (*n* = 85; 1.3%) and Fahal, A.H. (*n* = 55; 0.8%). Authors on the list were mainly from Egypt, Sudan, and Tunisia. Mapping research collaborations among researchers with a minimum of 20 publications (Fig. 7) showed that active authors (*n* = 55) were distributed into 16 clusters with only four clusters having five researchers or more. The maps also showed weak research collaboration between active researchers as shown by the absence of thin connecting lines.

**Table 6:** Top 10 active authors based in Arab countries and published in the field of NTDs

Author Name	Number of documents	% (*N* = 6542)	Country Affiliation
El-Hassan, A.M	86	1.3	Sudan
Morsy, T.A	85	1.3	Egypt
Fahal, A.H	79	1.2	Sudan
Aoun, K	51	0.8	Tunisia
El Ridi, R	51	0.8	Egypt
Khalil, E.A.G	46	0.7	Sudan
Farid, Z	45	0.7	Egypt/USA
Babba, H	41	0.6	Tunisia
Rondelaud, D	40	0.6	France
Bouratbine, A	38	0.6	Tunisia

## Discussion

In the current study, the contribution of researchers from the Arab region to scientific knowledge about NTDs in the past 50 years was presented. The findings of the study revealed relatively inadequate contribution given the population size and the presence of multiple risk factors for the emergence and spread of NTDs. Furthermore, the annual growth of publications from the Arab region remained slow and showed an observable rise only in the last year of the study period. The bulk of research from the Arab region focused on schistosomiasis and leishmaniasis with Egyptian authors and institutions making the greatest contribution to the field. However, the annual growth of publications from Saudi Arabia showed the steepest growth in the last decade. Extensive research collaboration was noted between researchers in Egypt and those in the US.

In the current study, the number of scientific publications authored/co-authored by researchers in the Arab region remained fluctuating and slowly increasing until the last year of the study period. Several reasons could be cited for the late and modest growth in the field. First, the call made by the WHO in 2012 through the NTD roadmap to increase efforts for the elimination and control of 10 NTDs by 2020 [38]. Second, the adoption of the SDGs in 2016 that called for the prevention and treatment of NTDs. The SDGs played a positive role in the growth of publications noted in the last few years of the study period. Third, the molecular advancement in the understanding of diseases also played a positive role in the growth of publications seen toward the end of the study period. Finally, the emergence of large numbers of refugees in the Arab region during the Arab Spring was a risk factor for the emergence of NTDs among refuges with limited health services. All these factors helped in the surge in the number of publications on NTDs from the Arab region seen lately.

The current study showed that researchers from 22 different Arab countries contributed to the scientific knowledge base on NTDs. This suggests that the negative health impact of NTDs is present all over the Arab region. In the Arab region, Egypt made the most significant contribution. There are several reasons for Egypt to rank first in the Arab region in the number of publications on NTDs. First, Egypt has the highest rates of NTDs in the Middle East and North Africa region [39]. Second, Egypt has a high prevalence of schistosomiasis [40], ascariasis, hookworm [41], and fascioliasis [42]. Third, Egypt with a population size of approximately 100 million has the highest number of academic and research institutions relative to other Arab countries. Fourth, the presence of specialized scientific associations and peer-reviewed scientific journals published by academic and medical Egyptian organizations helped in increasing the volume of publications from Egypt.

Analysis showed that Saudi Arabia ranked second in the number of publications. Saudi Arabia has a high prevalence of leishmaniasis, hookworm, and lymphatic filariasis [13]. The annual growth of publications from Saudi Arabia showed a steeper rise compared to that from Egypt. Reasons behind this include the relatively high income that enables research funding for academic and research institutions. A second potential reason for the ranking of Saudi Arabia next to Egypt is the large and extended geographical area with proximity to African tropical countries, Yemen, as well as countries in the South Eastern Asian countries. A third potential reason is the mass gatherings during *Hajj* and *Umrah* where approximately four million Muslims gather annually in a small region that facilitate the spread of infectious diseases [14]. Sudan ranked among the top three countries in the field. Sudan is still endemic for cutaneous and visceral leishmaniasis, as well as blinding trachoma [43].

The current study indicated that more than half of the publications were related to schistosomiasis, leishmaniasis, and echinococcosis. Both intestinal and urogenital schistosomiasis are prevalent in Africa and the Middle East and known for a long time. Schistosomiasis affects almost 240 million people worldwide, and more than 700 million people live in endemic areas [44]. The WHO strategy for schistosomiasis control focuses on preventive chemotherapy using praziquantel for affected populations. The Merck-WHO praziquantel donation program facilitated this strategy. In 2015 alone, 66.5 million treatments were donated. Schistosomiasis control has been successfully implemented in several countries, including Egypt, Oman, Jordan, Saudi Arabia, Morocco, and Tunisia [44]. The contribution of authors from the Arab region to the field of schistosomiasis was approximately 10% (data not shown) of the global research output on schistosomiasis indicative of active role of researchers in the region to the field of schistosomiasis. However, more efforts are still needed in the field of schistosomiasis. A major challenge in the field of schistosomiasis is the lack of complete understanding and appreciation by health policy makers to the actual disease burden and health consequences of schistosomiasis [45]. A second challenge in the field is the insensitivity of conventional diagnostic tools and inability of low- and middle-income countries to afford for high and expensive techniques such as the PCR [46]. Continuous screening of school children and aggressive treatment campaigns may help in reducing the prevalence rates of schistosomiasis [45].

Leishmaniasis was one of the most frequently researched NTDs by researchers in the Arab region. There are three main forms of the disease: cutaneous, visceral, and mucocutaneous leishmaniasis. The cutaneous form is the most common while the visceral is the most severe form while the mucocutaneous is the most disabling form [47]. According to the WHO fact sheet, in 2018, 92 and 83 countries or territories were considered endemic for or had previously reported cases of cutaneous and visceral leishmaniosis respectively. The current study showed that research on echinococcosis from Arab countries ranked third. The two most important forms, which are of medical and public health relevance in humans, are cystic echinococcosis, also known as hydatid disease or hydatidosis, and alveolar echinococcosis. Hydatidosis is endemic in sheep and cattle raising areas and its prevalence is very high in the Mediterranean region [48, 49]. A very recent study from Lebanon indicated that from 2018 to 2020, 62.9% of the sheep and 20.9% of the goats were found positive for cystic echinococcosis [50].

The current study indicated that STH was an under-researched topic relative to the high prevalence of STH in Arab countries. Approximately 1.5 billion people are infected with soil-transmitted helminths worldwide [51]. According to the WHO, Iraq, Somalia, Djibouti, Sudan, Syria, and Yemen are endemic areas and require interventions [52]. A study on STH in the Middle East and North Africa (MENA) region indicated that STH is widespread in the MENA region and a major reason for the widespread STH infections is the near absence of deworming in school-aged children [53]. Countries in the Arab region are still behind in the field of STH despite international recommendation to achieve the 2020 goal of STH control and elimination [54, 55].

The current study has a few limitations. There are several health-related journals produced by academic and research institutions in the Arab region and are not indexed in Scopus. Therefore, a certain number of documents on NTDs published in these unindexed journals were missed. Furthermore, despite the keywords used being comprehensive, certain documents might be missed because of the use of the title search strategy rather than the title/abstract/keyword strategy. The use of the title search strategy minimized the number of false-positive results and therefore it was used instead of the title/abstract/keyword strategy.

## Conclusions

The NTDs have a heavy economic and health burden on the affected regions. Arab countries cannot achieve SDG-03 goal by 2030 without the elimination and control of NTDs. The WHO has set ambitious targets in the road map for NTDs 2021 – 2030 [4, 56]. One of these ambitious targets is achieving a 90% reduction in the number of people in need of treatment against NTDs. Scientific advancement and research to fill in the knowledge gap is one of the approaches described by the WHO to achieve the targets in the roadmap for NTDs 2021 – 2030 [56]. The current study provided an analysis of the contribution of researchers from Arab countries to the knowledge base on NTDs using bibliometric indicators. The analysis revealed suboptimum contributions relative to population size and the extent of poverty in the Arab region. However, the last few years witnessed an increasing interest of researchers in Arab countries in NTDs. If the increase in the number of publications continues in the same pattern, researchers in the Arab countries will have a significant contribution to the field in the future. Researchers from Arab countries started their publications in the field utilizing specialized journals affiliated with institutions in the Arab region. However, with time, researchers from Arab countries targeted international and prestigious journals to disseminate their research. The research from Arab countries in the field of NTDs focused on three main diseases, specifically, schistosomiasis, leishmaniasis, and onchocerciasis. Soil-transmitted helminthiasis infection was under-researched despite its high prevalence in the Arab region. Several non-Arab countries, most notably the US, collaborated with Arab countries in this field. Analysis revealed many research networks with a minimum of 10 researchers. However, the connecting lines between clusters and within the same cluster were thin indicative of limited collaboration. Research collaboration among countries in the Arab region is needed to facilitate the exchange of knowledge and experience. More research on STH should be encouraged and supported. More research is also needed to cover the relationship between NTDs and various social, economic, and environmental aspects.

To achieve and implement the recommendations stated above to control and eliminate NTDs, the following strategies and suggestions need to be considered by Governments in the Arabic region:

1. Governments in the Arabic region should emphasize and increase the awareness of veterinary public health since several NTDs are of veterinary origin.
2. Governments need to make full access to medications used in chemoprevention and treatment of various NTDs. In this regard, pharmaceutical companies in the Arab region need to invest and manufacture medications related to NTDs
3. Governments and non-profit organizations should prioritize funding for molecular, pharmacological, and clinical research related to NTDs.
4. Research collaboration between pharmaceutical industry and academic institutions need to strengthened in the field of NTDs
5. Governments should allocate financial and non-financial prizes to scholars in the Arab region who made the most scientific contribution to the field of NTDs.
6. Governmental and non-governmental medical institutions should make available all up to date technology related to diagnosis of NTDs
7. Researchers in the field of microbiology/immunology should focus their efforts on the detection and prevalence of various NTDs especially in remote and urban areas to have a baseline data on the national status regarding various diseases.
8. Governments and academic institutions, especially in Egypt, need to provide scholarships for candidate graduate students to purse their investigations and molecular research on schistosomiasis in leading institutions in the US and the UK.
9. Governments need to establish national research centers for NTDs specially for schistosomiasis and leishmaniasis. Such national centers should be equipped with modern instruments and techniques to help recruit national and international scholars to do research and graduate studies in the center.
10. The Arab League should have special budget donated from governments and pharmaceutical companied dedicated for research on NTDs.
11. Academic institutions in the Arab region should organize annual scientific conference on NTDs research to help strengthen communication and collaboration among researchers in the field.
12. Academic institutions and pharmaceutical companies should support researchers in the field of NTD by covering for publication fees in prestigious journals.
13. Scholar in the Arab region should develop new research agendas on NTDs, especially on schistosomiasis, to fill in research gap in this field.
14. New research agendas in the field of NTDs need to be developed based on national and international communication among scholars in the field.

## Supplementary Information

**Additional file 1: Supplementary Material 1. **List and geographic location of Arab countries.  

**Additional file 2:** **Supplementary Material 2.** Search strategy and keywords used for the 20.  

## Data Availability

all data presented in this manuscript are available on the Scopus database using the search query listed in the methodology section.

## Abbreviations

WHO: World Health Organization
NTDs: Neglected Tropical Diseases
