# Supplementary material for: Contribution of researchers in Arab countries to scientific publications on neglected tropical diseases (1971 – 2020)
Source: Trop Dis Travel Med Vaccines. 2022 Jun 1;8:14. doi: 10.1186/s40794-022-00173-7 (PMC9159044; doi:10.1186/s40794-022-00173-7)
Supplement: Supplementary file 1 — Additional file 1: Supplementary Material 1. List and geographic location of Arab countries. [file 40794_2022_173_MOESM1_ESM.docx]

**Manuscript title: Contribution of researchers in Arab countries to research publications on Neglected Tropical Diseases (2001 – 2020)**

**Appendix 1: List of Arab countries (arranged alphabetically) and their population size [1]**

| **Country** | **Population (*10^3^)** |
| --- | --- |
| Comoros | 907.419 |
| Djibouti | 1016.097 |
| Bahrain | 1783.983 |
| Qatar | 2979.915 |
| Kuwait | 4380.326 |
| Mauritania | 4901.981 |
| Oman | 5323.993 |
| Palestine | 5345.541 |
| Lebanon | 6684.849 |
| Libya | 7040.745 |
| United Arab Emirates | 10081.785 |
| Jordan | 10300.869 |
| Tunisia | 12046.656 |
| Somalia | 16841.795 |
| Syria | 19364.809 |
| Yemen | 31154.867 |
| Saudi Arabia | 35844.909 |
| Morocco | 37772.756 |
| Iraq | 42164.965 |
| Algeria | 45350.148 |
| Sudan | 45992.020 |
| Egypt | 106156.692 |

**Geographical location of Arab countries (green color) in the world map**

**
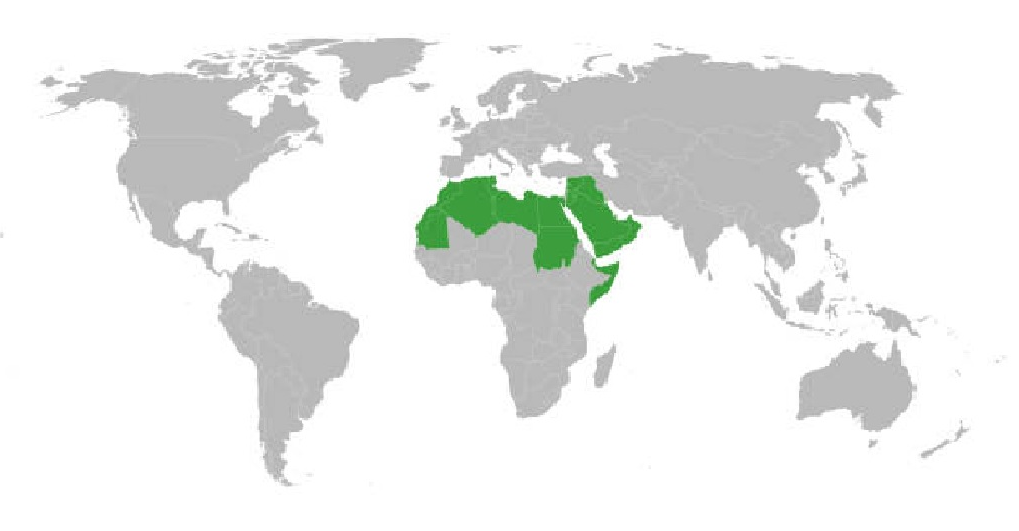
**

1. World Population Review: **Arab countries** Available from: <https://worldpopulationreview.com/country-rankings/arab-countries>. Accessed April 21, 2022.
