# Supplementary material for: Contribution of researchers in Arab countries to scientific publications on neglected tropical diseases (1971 – 2020)
Source: Trop Dis Travel Med Vaccines. 2022 Jun 1;8:14. doi: 10.1186/s40794-022-00173-7 (PMC9159044; doi:10.1186/s40794-022-00173-7)
Supplement: Supplementary file 2 — Additional file 2: Supplementary Material 2. Search strategy and keywords used for the 20. [file 40794_2022_173_MOESM2_ESM.docx]

**Appendix 2 Keywords and search strategy used in the study.**

**The keywords were obtained from a previously published study on neglected tropical diseases [1]**

| **Number** | **Name of the disease** | **Search query in Scopus** |
| --- | --- | --- |
| **1** | **Echinococcosis** | title ( ( echinococcosis ) or ( "cystic echinococcosis" ) or ( "alveolar echinococcosis" ) or ( "echinococcus granulosus" ) or ( "echinococcus multilocularis" ) or ( "polycystic echinococcosis" ) or ( "echinococcus vogeli" ) or ( "echinococcus oligarthrus" ) or ( "echinococcus canadensis" ) or ( "echinococcus granulosus sensu lato" ) or ( hydatidosis ) or ( "hydatid disease" ) or ( "alveolar hydatid cyst" ) ) |
| **2** | **Lymphatic filariasis** | title ( ( "lymphatic filariasis" or "wuchereria bancrofti" or "brugia malayi" or "brugia timori" or "elephantiasis" or "elephantiasis, filarial" or "filarial elephantiasis" or "bancroftian elephantiasis" or "elephantiases" or "filarial elephantiases" or "filariasis, lymphatic" or "filariases, lymphatic" or "lymphatic filariases" or "brugia malayi" ) ) |
| **3** | **Onchocerciasis** | title ( ( "onchocerciasis" or "river blindness" or "robles disease" or "onchocerca volvulus" or "erisipela de lacosta" or "mal morando" or "sowda" or "ocular onchocercias*" or "ocular onchocerciasis" ) ) |
| **4** | **Trachoma** | title ( ( "trachoma" or "chlamydia trachomatis" or "granular conjunctivitis" or "blinding trachmoa" or "trichiasis" or "egyptian ophthalmia" or "ophthalmia, egyptian" or "conjunctivitis - granular" ) ) |
| **5** | **Schistosomiasis** | title ( ( ( "schistosomiasis" or "bilharzia*" or "schistosoma mansoni" or "schistosoma haematobium" or "schistosoma japonicum" or "katayama fever" or "schistosoma haematobia" or "s. haematobium" or "schistosoma mansoni" or "s. mansoni" or "s. japoni*" or "s indicum" or "s. guineensis" or "s. intercalatum" or "schistosoma mekongi" or "s. mekongi" or "neuroschistosomiasis" or "bilharziasis" or "bilharziases" or "schistosomiases" or "schistosomiasis, urinary" or "urinary schistosomiasis" or "schistosomiasis, intestinal" or "intestinal schistosomiases" or "intestinal schistosomiasis" or "schistosomiases, intestinal" or "neuroschistosomiases" or "central nervous systemschistosomiasis" or "schistosomiasis, central nervous system" or "schistosomalmyelopathy" or "myelopathies, schistosomal" or "myelopathy, schistosomal" or "schistosomal myelopathies" or "schistosomal myeloradiculopathy" or "myeloradiculopathies, schistosomal" or "myeloradiculopathy, schistosomal" or "schistosomal myeloradiculopathies" or "schistosomal myelitis" or "myelitis, schistosomal" or "schistosoma" or "schistosomes" ) ) ) |
| **6** | **Buruli ulcer** | title ( ( "buruli ulcer*" or "mycobacterium ulcerans" or "infection, mycobacterium ulcerans" or "ulcer, buruli" ) ) |
| **7** | **Chagas disease** | title ( ( "chagas disease" or "american trypanosomiasis" or "trypanosoma cruzi" or "triatominae" or "kissing bugs" or "t. cruzi infection" or "south american trypanosomiasis" or "disease, chagas" or "trypanosomiasis, american" or "trypanosomiasis, south american" or "chagas' disease" or "trypanosoma cruzi" ) ) |
| **8** | **Mycetoma** | title ( ( "chromoblastomycosis" or "fonsecaea pedrosoi" or "cladophialophora carrionii" or "phialophora verrucosa" or "fonsecaea compacta" or "mycetoma" or "mycetoma belt" or "deep mycoses" ) ) or |
| **9** | **Taenia solium** | title ( ( "cysticercosis" or "taenia solium" or "taeniasis" or "pork tapeworm" or "human cysticercosis" or "neurocysticercosis" or "t. solium" or "taeniasis/cysticercosis" ) ) |
| **10** | **Dengue fever and chikungunya** | title ( "dengue fever" or "dengue" or "dengue virus" or "breakbone fever" or "dengue shock syndrome" or "chikungunya" or "chikungunya virus" or "chikv" ) |
| **11** | **Dracunculiasis** | title ( "dracunculiasis*" or "guinea worm disease" or "guinea worm" or "dracunculus medinensis" or "guinea-worm" or "dracunculo*" or "dracontiasis" or "medina worm" or "dracunculiasis*" or "dracunculo*" or "dracontiasis" or "medina worm" or "dracunculiases" or "guinea worm infection" or "infection, guinea worm" ) |
| **12** | **Snakebite envenoming** | title ( "snakebite envenoming" or "snakebite disease" or "venomous snake" or "snake bite" ) |
| **13** | **Human African trypanosomiasis** | title ( "human african trypanosomiasis" or "african sleeping sickness" or "sleeping sickness" or "trypanosoma brucei" or "trypanosoma brucei gambiense" or "trypanosoma brucei rhodesiense" or "african trypanosomiasis*" or "trypanosomiasis, african" or "african trypanosomiases" or "trypanosomiases, african" or "sleeping sickness, african" or "nagana" or "trypanosomes" or "t. gambiense" or "t. rhodesiense" ) |
| **14** | **Leishmaniasis** | title ( "leishmaniasis" or "leishmania parasites" or "mucocutaneous leishmaniasis" or "leishmania infection" or "black fever" or "oriental sore" or "delhi boil" or "visceral leishmaniasis" or "cutaneous leishmaniasis" or "mucocutaneouso" or "leishmaniasis, visceral" or "kala-azar" or "kala azar" or "cutaneous leishmaniases" or "leishmaniases, cutaneous" or "sore, oriental" or "leishmaniasis, old world" or "old world leishmaniasis" or "leishmaniasis, new world" or "new world leishmaniasis" or "leishmaniasis, american" or "american leishmaniasis" or "l.donovani" or "l.infantum" or "l.chagasi" or "l.mexicana" or "l.amazonensis" or "l.venezuelensis" or "l.tropica" or "l.major" or "l.aethiopica" or "l.v. braziliensis" or "l. v. guyanensis" or "l. v. panamensis" or "l.v. peruviana" ) |
| **15** | **Foodborne trematodiases** | title ( "foodborne trematodiases" or "clonorchis" or "opisthorchis" or "fasciola" or "paragonimus" or "trematodes" or "clonorchis sinensis" or " opisthorchis viverrini" or "fishborne trematodes" or "paragonimus westermani" or "paragonimus heterotremus" or "paragonimus philippinensis" or "fasciola hepatica" or "fasciola gigantica" ) |
| **16** | **Leprosy** | title ( "leprosy" or "hansen's disease" or "mycobacterium leprae" or "leprosy tuberculoid" or "leprosy, neural" or "leprosy, lepromatous" or "leprosy, cutaneous" or "leprosy modular" or "leprosy, borderline" or "leprosy dimorphous" or "leprosy histoid" ) |
| **17** | **Rabies** | title ( "rabies" or "furious rabies" or "paralytic rabies" or "rabies virus" or "dog-mediated human rabies" or "dog-mediated rabies" ) |
| **18** | **Scabies** | title ( "scabies" or "sarcoptes scabiei var hominis" or "human itch mite" or "crusted scabies" or "norwegian scabies" or "sarcoptes scabiei" or "human scabies" or "tungiasis" or "tunga penetrans" or "t. trimamillata" or "pulga de areia" or "nigua" or "pique" or "bicho do pé" or "bichodo porco" or "jatecuba" or "jigger" or "sand flea" or "chigoe" ) |
| **19** | **Soil-transmitted helminthiases** | title ( "soil-transmitted helminthiases" or "soil-transmitted helminth" or "ascaris lumbricoides" or "trichuris trichiura" or "necator americanus" or "ancylostoma duodenale" or "ascaris" or "whipworm" or "hookworm" or "ascariasis*" or "parasitic roundworm disease" or "a.lumbricoide*" or "trichocephalus trichiuris" or "trichuriasis" or "human whipworm" or "trichocephaliasis" or "trichocephaliases" or "whipworm disease" or "new world hookworm" or "n. americanus" or "old world hookworm" or "hookworm infection" ) |
| **20** | **Yaws** | ( "yaws" or "endemic treponematoses" or "treponema pertenue" or "secondary yaws" or "human yaws" or "pertenue" ) |
| **21** | **General** | title ( ( "neglected tropical diseases" or "ntd" or "ntds" or "neglected tropical disease" ) ) ) |
| **22** | **Country affiliation** | ( affilcountry ( jordan or syria* or lebanon or egypt or iraq or sudan or kuwait or saudi or emirates or "united arab" or yemen or oman or bahrain or qatar or morocco or algeria or mauritania or comoros or palestine or gaza or djibouti or tunisia or libya* or somalia ) ) |
| **23** | **Study period** | 1971 - 2020 |
| **24** | **Source type** | journal documents |
| **25** | **Overall search query** | 1 – 21 research queries were connected with "OR" Boolean operator. The net result was connected with queries #22, #23, and #24 with the Boolean operator "AND". |
|  |  |  |

1. Gyorkos TW, Carabin H, Phillip M, et al. Canadian contributions to research on neglected tropical diseases. PLoS neglected tropical diseases. 2021;15(7):e0009476.
